# Finding the Primary Care Providers in the Specialist-Dominant Primary Care Setting of Korea: A Cluster Analysis

**DOI:** 10.1371/journal.pone.0161937

**Published:** 2016-08-25

**Authors:** Jin Yong Lee, Sang Jun Eun, Hyun Joo Kim, Min-Woo Jo

**Affiliations:** 1 Public Health Medical Service, Boramae Medical Center, Seoul National University College of Medicine, Seoul, Korea; 2 Department of Preventive Medicine, Chungnam National University School of Medicine, Daejeon, Korea; 3 Department of Nursing Science, Shinsung University, Dangjin, Chungnam, Korea; 4 Department of Preventive Medicine, University of Ulsan College of Medicine, Seoul, Korea; Johns Hopkins University Bloomberg School of Public Health, UNITED STATES

## Abstract

**Objective:**

This study aimed to identify private clinics that have a potential to perform the role of primary care providers (PCPs) in a primary care setting in Korea where private specialists are dominant.

**Methods:**

The 2013 National Patient Sample claim data of Health Insurance Review and Assessment Service in Korea was used. Two-step cluster analysis was performed using characteristics of private clinics, and patient and utilization characteristics of 27,797 private clinics. External validation of clusters was performed by assessing the association among clusters and outcomes of care provided by private clinics. Stability of clusters was cross-validated using discriminant analysis.

**Results:**

The result classified more than a half of private clinics into a potential PCP cluster. These were private clinics with specialties considered to be those of primary care physicians and were more likely to be located in non-metropolitan areas than specialized PCPs were. Compared to specialized PCPs, they had a higher percentage of pediatric and geriatric patients, patients with greater disease severity, a higher percentage of patients with complex comorbidities or with simple or minor disease groups, a higher number of patients and visits, and the same or higher quality of primary care. The most important factor in explaining variations between PCP clusters was the number of simple or minor disease groups per patient.

**Conclusion:**

This study identified potential PCPs and suggested the identifying criteria for PCPs. It will provide useful information for formulation of a primary care strengthening policy to policy makers in Korea as well as other countries with similar specialist-dominant primary care settings.

## Introduction

Primary care is widely regarded as a core component of a rational healthcare system [1–3]. This is because a strong primary care system could improve the effectiveness, efficiency, and equity of the healthcare system, thereby reducing mortality rates, providing optimal preventive care, reducing unnecessary hospitalization and emergency room admissions, improving quality of life and outcomes, and increasing access to health services for relatively deprived population groups [4–6]. However, the community-based primary care sector in the Republic of Korea (hereafter referred to as Korea) is underdeveloped [7]. Also, the number of potentially avoidable admissions for chronic obstructive pulmonary disease (COPD), asthma, and uncontrolled diabetes is amongst the highest in the Organization for Economic Cooperation and Development (OECD) countries [7, 8]. Therefore, developing the primary care system has been a major challenging and high-priority task for improvement of Korea’s healthcare system [7].

The Korean healthcare system is dominated by the private sector, which represents more than 90% of all existing medical care institutions [9, 10]. Patients can visit any specialty clinic or hospital without referrals from regular family physicians since the healthcare delivery system has no gatekeeping function and is poorly differentiated [11–13]. All doctors can operate a self-owned private clinic irrespective of their medical specialty [11], and 92.6% of physicians in private clinics are qualified specialists [14]. Further, since compensation is based on a fee-for-service payment system, physicians lack incentives to provide disease prevention and health promotion services [10].

The government of Korea has made several attempts to establish a healthcare delivery system and to strengthen primary care, including the introduction of a preferred doctor system as a regular source of care. However, these attempts have not been successful because of resistance from professional medical societies, insufficient commitment from the government, and paucity of evidence supporting the applicability of such a system in Korea [10, 11, 15, 16].

For successful implementation of a primary care improvement policy, various obstacles need to be overcome by applying solutions such as building an incentive scheme for acceptance of the policy by the stakeholders such as patients and healthcare providers, assessment of the effectiveness of the policy, and assessment of the quality of primary care services [17]. However, the specialist who will play the role of the primary care provider (PCP) needs to be defined. This is because in the specialist-dominant primary care setting of Korea, physicians who are systemically trained to provide primary care are lacking and hence it would be difficult to provide high quality primary care unless qualified PCPs are trained [18]. In addition, it would be difficult to select a certain specialty to be classified as the PCP in a political and practical sense. In the US, general internists, general pediatricians, and family physicians are considered as primary care physicians, but there has also been controversy regarding the need to classify obstetricians, gynecologists, and general surgeons as primary care physicians [19, 20]. Similarly, in Korea, internists, pediatricians, family physicians, and general practitioners are regarded as primary care physicians based on the assumption that they have the ability to provide comprehensive care; however, there is controversy regarding whether other specialists, such as general surgeons, obstetrician-gynecologists, and oriental medicine doctors, are considered primary care physicians [19]. As Korea currently harbors a competitive medical market in which patients have almost no limitations in choosing medical institutions, the medical practitioners that have specialties other than those classified as primary care physicians have been opposing their exclusion from PCPs because implementation of the preferred doctor system might make it difficult for them to secure patients [17]. Further, even among specialists considered to be primary care physicians, some provide profit-driven medical services such as services not covered by medical insurance, which is distinct from the role of primary care providers [21, 22]. Finally, even in an attempt to evaluate the effectiveness of a primary care strengthening policy and the quality of primary care, only a conceptual definition of PCPs exists and realistic policy targets cannot be clearly defined [16, 18, 23]. As such, an unclear definition of what kind of PCP can and should provide primary care is a practical barrier to implementation of a primary care strengthening policy in Korea and for the accumulation of supporting evidence.

A PCP should be a provider who appropriately performs specific functions of primary care. In Korea, concepts of primary care consist of 4 core attributes of first contact, comprehensiveness, coordination of care, and longitudinality and 3 ancillary attributes of personalized care, context of family and community, and community-based care [24]. The Korean primary care assessment tool (K-PCAT) was developed in order to measure the following attributes of primary care: comprehensiveness, coordination, personalized care, family and community orientation, and first contact [11, 25]. However, as the K-PCAT assesses the quality of primary care practice based on the patient’s experience [11, 19, 25, 26], the data collection is time consuming and expensive. Further, as PCPs with good performance are rare and overall primary care quality is poor in Korea, opportunities are limited for identifying adequate PCPs and utilizing them for the training scheme. Comprehensiveness is a common element in many definitions of primary care [27]. One of the roles of primary care is to act as the gateway to the healthcare delivery system, in addition to addressing the “common healthcare needs of people” [24]. This is required to provide continuity and coordination of care in the speciality-dominant care setting of Korea, in which patients are allowed unlimited choices of health care providers. In a setting where a patient selects a specialty clinic based on self-assessed needs, the patient may have to choose another clinic for health problems that could not be addressed at the first clinic. Thus, a provider who can comprehensively manage most health problems of a patient will enable continuity and coordination of care.

Therefore, this study aimed to identify potential PCPs and their characteristics through cluster analysis using characteristics of private clinics, and patient and utilization characteristics of private clinics, while focusing on the comprehensiveness attributes of primary care.

## Materials and Methods

### Data sources

The 2013 National Patient Sample (NPS) claim data of Health Insurance Review and Assessment Service (HIRA) was utilized (serial number: HIRA-NPS-2013-0084). The HIRA-NPS data is an anonymous stratified random sample of claims data of HIRA for 3% of the entire population (approximately 1.4 million patients per year), consisting of 10% inpatients and 90% outpatients [28]. The Patient Sample was obtained using a stratified sampling, a probabilistic sample extraction method [28]. The sample was divided into 32 strata based on sex (2 strata) and age (16 strata with 5-year interval) before random extraction [28]. Stratification at the patient level secures the representativeness of the claims data by accounting for differences in healthcare service settings (inpatient or outpatient), the cycle of claims data submissions from providers (daily or monthly), and disease types [28]. As the claim cost in the National Health Insurance claims data exhibits the maximum variance and best reflects the characteristics of the claims data, claim cost was chosen as a sample variable [28]. Assuming a normal distribution and an acceptable sampling error range, the standard deviation and sample size were calculated. A sample size that was most representative of the overall claims data was then determined [28]. There was a 95% concordance between the estimated population (45,861,321 persons) and the actual population (47,026,505 persons), exhibiting a high level of representativeness [28]. The database comprises of a total of 5 relational tables: Table 20 (general specifications), Table 30 (health services), Table 40 (diagnosis information), Table 53 (outpatient prescription), and Table of Providers (healthcare service provider information) [28]. The strengths of the HIRA-NPS data are their representativeness, verified validity allowing for generalization for the population, and a comprehensive information set covering all services provided under the fee-for-service payment system [28, 29]. HIRA’s Patient Sample data can be purchased and used by filling out the End User Agreement of the Patient Samples form on the ‘Healthcare Bigdata Hub’ site (http://opendata.hira.or.kr/op/opc/selectPatDataAplInfoView.do). The 2013 HIRA-NPS data include 22,344,539 claims, of which 12,553,133 were outpatient claims showing no history of admission and were from primary care institutions not hospitals. To identify PCPs, data in the unit of claims were aggregated based on different private clinics. The sum of the number of patients for each clinic was 3,304,445 (applying 33.3 of sample weights, it was 110,147,042) and the number of private clinics was 27,797.

### Measures

Unit of analysis for the study was the level of private clinic and all variables were extracted as annual numbers or proportions of a clinic.

#### Characteristics of private clinics

Characteristics of private clinics were specialty and location of clinic. The Table of Providers in the HIRA-NPS data contains information about healthcare providers but not about specialty of private clinic owners or specialty of clinics. Therefore, a specialty variable was generated based on the method used by HIRA to classify the specialty of clinics. Clinic-level medical institutions are required to write down the specialty of the service provided for every claim when filing service claims to HIRA after providing service to patients. Owing to the high competitiveness of the medical market, there are some reports of physicians providing relatively more profitable services regardless of their specialty [21, 22]. Therefore, HIRA implemented the requirement in order to confirm specialty of the clinic. HIRA considers a clinic with over 50% of their claims in a particular specialty area as a specialty clinic in the particular area and those with less than 50% of specialty claims as a general practitioner. In this study, specialty is defined as self-assigned specialty following the same method. Locations of the clinics were 17 provinces that included metropolitan regions of Seoul, Busan, Incheon, Daegu, Gwangju, Daejeon, Ulsan, Sejong, and Gyeonggi and non-metropolitan regions of Gangwon, Chungbuk, Chungnam, Jeonbuk, Jeonnam, Gyeongbuk, Gyeongnam, and Jeju.

#### Patient characteristics of private clinics

Characteristic variables of clinic patients included the percentages of female patients, age ≤19 years, age ≥65 years, and Medical Aid of all patients at the clinic in a year. To reflect the severity of the outpatient patients, proportion of patients with Charlson comorbidity index (CCI) score of ≥1 was used. The CCI is a well-validated and commonly used risk adjustment tool. CCI is a composite score calculated by summating the weighted relative risks of 1-year mortality of the 17 conditions. CCI was calculated using the Sundararajan version [30, 31].

To account for the comprehensiveness of diseases covered by clinics, the percentage of patient with simple or minor disease groups (SMDGs) was used. The Korean government designated 52 SMDGs in 2011, which have been recommended to be managed in primary care settings [32]. The 52 SMDGs are common minimal disease groups in Koreans, which can be and are preferably resolved by primary care; the SMDGs were selected by consensus after five meetings with stakeholders, including providers (Korean Medical Association and Korean Hospital Association), academic societies (Korean Academy of Medical Sciences and various societies of medical specialties), government (Ministry of Health and Welfare and HIRA), and patient interest groups [33]. In October 2011, the National Health Insurance Act introduced the Special Provisions Concerning Individual Co-payment Calculation to redirect the flow of patients with SMDGs from hospitals to primary practices; prescription drug cost-sharing increased from 30% to 50%, or to 40% in case wherein tertiary or general hospital outpatient services were utilized for treating SMDGs [32, 34]. Table 1 lists the 52 SMDGs’ International Classification of Diseases, Tenth Revision (ICD-10) codes, and associated International Classification of Primary Care, Second Edition, Electronic (ICPC-2E) codes. Up to 10 diagnosis codes that required the most resources at each visit were identified, and if any of the 10 diagnoses corresponded with the SMDGs codes, the visit was regarded as a visit for SMDGs. Among all of the claims that a patient had filed in a year, if a patient had a visit for an SMDG, the patient was considered to be a patient with SMDGs. Further, to account for multiple comorbidities, the number of SMDGs per patient per clinic was used.

**Table 1:** ICD-10 codes of SMDGs and related ICPC-2E codes.

No	SMDGs	ICD-10 codes	Related ICPC-2E codes
1	Other gastroenteritis and colitis of infectious and unspecified origin	A09.0-A09.9	D73
2	Dermatophytosis	B35.2-B35.6, B35.8, B35.9	S74
3	Non-insulin-dependent diabetes mellitus	E11.2-E11.9	T90
4	Disorders of lipoprotein metabolism and other lipidemias	E78.0-E78.9	T93
5	Hordeolum and chalazion	H00.0, H00.1	F72
6	Disorders of lacrimal system	H04.0-H04.9	F03, F73, F99
7	Conjunctivitis	H10.0-H10.9	F70, F71
8	Senile cataract	H25.0-H25.9	F92
9	Disorders of refraction and accommodation	H52.0-H52.7	F91
10	Otitis externa	H60.1, H60.3, H60.5, H60.8, H60.9	H70
11	Essential hypertension	I10.0, I10.9	K86
12	Acute nasopharyngitis	J00	R74
13	Acute sinusitis	J01.0-J01.9	R75
14	Acute pharyngitis	J02.0-J02.9	R72, R74
15	Acute tonsillitis	J03.0-J03.9	R72, R76
16	Acute laryngitis and tracheitis	J04.0-J04.2	R77
17	Acute upper respiratory infections of multiple and unspecified sites	J06.0-J06.9	R74
18	Acute bronchitis	J20.9	R78
19	Vasomotor and allergic rhinitis	J30.0-J30.4	R97
20	Chronic nasopharyngitis and pharyngitis	J31.1, J31.2	R83
21	Chronic sinusitis	J32.0-J32.9	R75
22	Asthma	J45.0-J45.9	R96
23	Gastro-esophageal reflux disease	K21.0-K21.9	D84
24	Gastric ulcer	K25.3, K25.7, K25.9	D86
25	Peptic ulcer, site unspecified	K27.3, K27.7, K27.9	D86
26	Gastritis and duodenitis	K29.0-K29.9	D87
27	Dyspepsia	K30	D07
28	Other noninfective gastroenteritis and colitis	K52.2, K52.3, K52.8, K52.9	D11, D99
29	Irritable bowel syndrome	K58.0-K58.9	D93
30	Other functional intestinal disorders	K59.0-K59.2, K59.4, K59.8, K59.9	D04, D11, D12, D99
31	Other diseases of liver	K76.0, K76.9	D97
32	Atopic dermatitis	L20.8, L20.9	S87
33	Allergic contact dermatitis	L23.8, L23.9	S88
34	Urticaria	L50.0-L50.9	S98
35	Other arthritis	M13.0-M13.9	L91
36	Spondylosis	M47.8, M47.9	L83, L84
37	Cervical disc disorders	M50.9	L83
38	Other intervertebral disc disorders	M51.3, M51.4, M51.8, M51.9	L84, L86
39	Dorsalgia	M54.8, M54.9	L02
40	Synovitis and tenosynovitis	M65.2, M65.3, M65.8, M65.9	L87
41	Shoulder lesions	M75.0, M75.2, M75.9	L92
42	Other enthesopathies	M77.8, M77.9	L87
43	Other soft tissue disorders, not elsewhere classified	M79.1, M79.4, M79.6, M79.8, M79.9	L09, L12, L14, L17, L18, L19, L99
44	Osteoporosis without pathological fracture	M81.0-M81.9	L95
45	Cystitis	N30.0, N30.9	U71
46	Chronic prostatitis	N41.1	Y73
47	Other inflammation of vagina and vulva	N76.0, N76.2	X84
48	Menopausal and other perimenopausal disorders	N95.1, N95.2, N95.9	X11
49	Sprain and strain of joints and ligaments of lumbar spine and pelvis	S33.5-S33.7	L79, L84
50	Sprain and strain of joints and ligaments at wrist and hand level	S63.6, S63.7	L79
51	Sprain and strain of other and unspecified parts of knee	S83.6	L78
52	Sprain and strain of joints and ligaments at ankle and foot level	S93.5, S93.6	L79

Abbreviations: SMDGs, simple or minor disease groups; ICD-10, International Classification of Diseases, Tenth Revision; ICPC-2E, International Classification of Primary Care, Second Edition, Electronic.

#### Utilization characteristics of private clinics

Utilization characteristics of private clinics were the number of patients, number of patients with SMDGs, number of visits, and number of visits per patient per year. The proportion of visits to a specific clinic and the number of additional clinics visited were used as a proxy for comprehensiveness and continuity of care. The proportion of visits to a specific clinic was the average proportion of visits to a specific clinic by each patient, calculated by dividing the number of visits to a specific clinic of a patient by the total number of visits of a patient [35]. The number of additional clinics visited was the number of clinics visited other than a specific clinic. Cost was not used for clustering of private clinics for classification of PCPs but it was used for external validation of clusters: total claim cost, cost covered by National Health Insurance Service (NHIS), and out-of-pocket (OOP) cost, which is the patient’s cost share. Currency was converted to USD using 2013 conversion ratio between KRW and USD (1 USD = 1,055.4 KRW).

#### Outcomes of care provided in private clinics

For external validation of clusters of private clinics, ambulatory care sensitive condition (ACSC) admission rates and length of stay (LOS) for ACSCs admission were used. ACSC admission rates were calculated according to the primary care area measures from Health Care Quality Indicators of OECD [36]. Indicators of primary care area were admission rates for 7 ACSCs: asthma, COPD, congestive heart failure (CHF), diabetes without complications, diabetes short-term complications, diabetes long-term complications, and diabetes lower extremity amputation [36]. Of these, admission rates of 6 ACSCs, excluding admission rate for diabetes lower extremity amputation, which could not be identified in the HIRA-NPS data, were used. With the number of patients with principal diagnosis codes of ACSC as the denominator and the number of all non-maternal/non-neonatal admissions with a principal diagnosis code of ACSC as the numerator [36], 6 admission rates based on ACSC and the total admission rates of 6 ACSCs for each private clinic were calculated. Patients aged <75 years were subjects of the study [37] and patients transferring from other institutions, having exclusion diagnosis codes and LOS of <1 day were excluded [36]. LOS for ACSCs admission was calculated as a total of LOS from 6 ACSCs admissions. To consider the severity of ACSCs, Poisson regression analysis was performed with ACSCs admission rates and LOS for ACSCs admission as dependent variables, and age, sex, and CCI score as independent variables. The observed-to-expected (O/E) ratios for ACSCs admission rates and LOS for ACSCs admission of each private clinic were calculated based on the results of Poisson regression analysis.

### Statistical analysis

#### Two-step cluster analysis

As the characteristics needed to identify private clinics subgroups include both categorical and continuous variables, two-step cluster analysis for SPSS was performed [38]. Two-step clustering method forms preclusters in the first step to reduce the size of the matrix that contains distances between all possible pairs of subjects, and in the second step, these preclusters are grouped into preferred number of clusters by using a hierarchical clustering algorithm (if the desired number of clusters is unknown, the SPSS two-step analysis automatically determines the proper number of clusters) [39, 40]. Before clustering the private clinics, factor analysis was performed to reduce the number of clustering variables. 52 proportions of patient with SMDGs that might be interrelated were reduced to 20 proportions of patient with simple or minor conditions (SMCs) by factor analysis: common disease in primary care (SMDGs’ serial number 3, 4, 11, 25, 26, 29, 31, 35, 39, 44 in Table 1), eye disease (5–9), skeletal disease (36–38, 40–42, 49–52), female disease (45, 47, 48), skin disease 1 (2, 33, 46), ear and nose disease (10, 19–21), gastroenteritis (1, 28), acute sinusitis (13), skin disease 2 (32, 34), dyspepsia (27), acute upper respiratory infection (17), acute laryngitis and tracheitis (16), acute nasopharyngitis (12), acute bronchitis (18), other soft tissue disorder (43), acute tonsilitis (15), other functional intestinal disorder (30), asthma and gastric ulcer (22, 24), acute pharyngitis (14), and gastro-esophageal reflux disease (23). Cronbach’s alpha was 0.617 for 20 proportions of patient with SMCs. The proportion of patient with SMCs was the average percentage of patient with SMDGs in each SMC category. Private clinics were clustered based on their similarity on 33 clustering variables: specialty and location of clinics (2 characteristics of private clinics), percentages of female patients, patients aged ≤19 years, patients aged ≥65 years, Medical Aid patients, patients with CCI score of ≥1, and patients with SMDGs, number of SMDGs per patient, 20 percentages of patients with each SMC (27 patient characteristics of private clinics), proportion of visits to specific clinic, number of additional clinics visited, number of patients with SMDGs, and number of visits per patient (4 utilization characteristics of private clinics). Log-likelihood criterion was used for distance measure, and continuous variables were standardized using z-scores. Akaike’s Information Criterion was applied to find proper number of clusters. Overall goodness-of-fit of clusters that were formed as results was evaluated using silhouette coefficient. Silhouette measure of less than 0.2 was classified as poor, between 0.2 and 0.5 as fair, and more than 0.5 as good solution quality [38], of which fair or higher was considered acceptable clustering.

#### Validation of the clusters

External and internal validations of two-step cluster solution were evaluated. External validation of clusters was performed by assessing the association among clusters and 6 ACSC admission rates and O/E ratios, all 6 ACSCs admission rate and O/E ratio, LOS for ACSCs admission and O/E ratio, total claim cost, NHIS cost, and OOP cost by using *t* test. To evaluate whether subjects were allocated to appropriate cluster groups generated based on private clinic characteristics, discriminant analysis was performed for internal validation [41, 42]. Total of 27,797 private clinics were randomly split into test sample with 13,787 clinics and holdout sample with 14,010 clinics. Then, discriminant analysis was performed using the test samples by entering all clustering variables into a model. After applying discriminant coefficient to holdout samples based on the results, predicted clusters, to which private clinics would belong, were calculated. Further, stability or replicability of the clusters was evaluated by calculating the percentage correctly classified between the predicted cluster based on discriminant analysis by using holdout sample and actual cluster based on two-step cluster analysis [42].

All the analyses were completed using SAS, version 9.3 (SAS Institute, Inc., Cary, NC) and SPSS, version 22 (IBM Corp., Armonk, NY). All statistical tests were two-sided and a p-value <0.05 was considered statistically significant.

### Ethics considerations

This study was approved by the Institutional Review Board of Chungnam National University School of Medicine (IRB No.15-05).

## Results

### General characteristics of private clinics

Total number of private clinics was 27,979, in which internal medicine was the most common specialty at 27.8%, followed by orthopedics, dermatology, general practitioners, pediatrics, and otorhinolaryngology. About 27.3% of all private clinics were located in non-metropolitan areas. About 58.0% patients visiting clinics were female, 19.2% were aged ≤19 years, 14.8% were aged ≥65 years, and 3.3% were Medical Aid beneficiaries, 9.1% were patients with CCI score of ≥1, and 77.8% were patients with SMDGs. Further, on average, a patient had 2.0 SMDGs. About 3,963 patients visited each clinic in a year on average, of which 3,311 patients had SMDGs. Average number of visits per year was 14,629, leading to average visits of 4.2 times per patient per year. Average proportion of visits to a specific clinic was 32.7% and average number of additional clinics visited was 3.5 (Table 2).

**Table 2:** Characteristics of the potential and specialized primary care provider clusters.

			Total	Potential PCP cluster	Specialized PCP cluster	p-value
Number of private clinics			27,797	14,710	13,087	
Characteristics of private clinics			N	%	%	
Specialty	Internal medicine		7,714	93.8	6.2	<0.001
	Family medicine		994	85.8	14.2	
	Pediatrics		1,858	99.5	0.5	
	General practitioner		2,320	70.2	29.8	
	Otorhinolaryngology		1,679	99.0	1.0	
	Thoracic and cardiovascular surgery		83	51.8	48.2	
	General surgery		963	43.5	56.5	
	Psychiatry		874	0.3	99.7	
	Neurology		94	17.0	83.0	
	Obstetrics and gynecology		1,390	0.6	99.4	
	Orthopedics		3,860	20.9	79.1	
	Neurosurgery		208	4.8	95.2	
	Plastic surgery		128	0.0	100.0	
	Ophthalmology		1,502	0.4	99.6	
	Anesthesiology		541	7.9	92.1	
	Rehabilitation medicine		135	25.9	74.1	
	Dermatology		2,369	0.8	99.2	
	Urology		924	2.9	97.1	
	Radiology		119	13.4	86.6	
	Other		42	69.0	31.0	
Location of clinics	Non-metropolitan area		7,579	56.6	43.4	<0.001
	Metropolitan area		20,218	51.5	48.5	
Patient characteristics of private clinics			Mean (SD)	Mean (SD)	Mean (SD)	
Proportion of female patients (%)			58.0 (15.6)	55.5 (8.9)	60.8 (20.3)	<0.001
Proportion of patients aged ≤19 (%)			19.2 (19.1)	24.3 (22.0)	13.3 (12.8)	<0.001
Proportion of patients aged ≥65 years (%)			14.8 (13.2)	15.2 (13.3)	14.4 (13.1)	<0.001
Proportion of Medical Aid patients (%)			3.3 (4.5)	3.4 (4.9)	3.3 (4.2)	0.062
Proportion of patient with CCI score of ≥1 (%)			9.1 (14.7)	15.4 (17.2)	2.0 (5.7)	<0.001
Proportion of patient with SMDGs (%)			77.8 (25.9)	92.9 (7.4)	60.9 (28.5)	<0.001
Number of SMDGs per patient			2.0 (1.2)	2.9 (0.9)	1.0 (0.6)	<0.001
Utilization characteristics of private clinics			Mean (SD)	Mean (SD)	Mean (SD)	
Number of patients			3,962.6 (3,509.8)	4,254.3 (3,328.8)	3,634.6 (3,675.1)	<0.001
Number of patient with SMDGs			3,310.8 (3,232.7)	3,981.7 (3,141.1)	2,556.7 (3,167.5)	<0.001
Number of visits			14,628.7 (11,692.6)	17,291.0 (11,665.5)	11,636.3 (10,977.9)	<0.001
Number of visits per patient			4.2 (4.7)	4.8 (6.0)	3.6 (2.2)	<0.001
Proportion of visits to a specific clinic			32.7 (8.9)	36.3 (8.0)	28.6 (8.1)	<0.001
Number of additional clinics visited			3.5 (0.7)	3.3 (0.6)	3.8 (0.8)	<0.001
Total claim cost (USD)			262,360.1 (574,836.1)	273,376.0 (647,117.6)	249,978.0 (480,513.1)	<0.001
NHIS cost (USD)			198,557.6 (506,743.4)	209,920.2 (574,783.2)	185,785.9 (416,877.4)	<0.001
OOP cost (USD)			63,802.4 (77,746.1)	63,455.8 (80,437.5)	64,192.1 (74.606.3)	0.429
Outcomes of care provided in private clinics			Mean (SD)	Mean (SD)	Mean (SD)	
All 6 ACSCs		Admission rate (%)	17.5 (60.2)	13.5 (46.6)	44.5 (113.0)	<0.001
		O/E ratio^*^	33.2 (797.8)	20.7 (123.9)	119.7 (2,214.6)	0.069
Asthma		Admission rate (%)	5.9 (26.0)	5.7 (25.6)	9.1 (32.0)	0.037
		O/E ratio	10.1 (45.6)	9.8 (45.2)	14.2 (52.5)	0.094
COPD		Admission rate (%)	9.5 (44.9)	9.6 (45.7)	8.6 (39.0)	0.681
		O/E ratio	65.7 (1,295.3)	59.5 (1,288.1)	110.9 (1,347.8)	0.452
CHF		Admission rate (%)	3.3 (17.0)	3.4 (17.2)	1.8 (13.2)	0.470
		O/E ratio	6.9 (44.3)	7.1 (45.1)	3.8 (28.3)	0.581
DM		Admission rate (%)	7.1 (37.4)	6.3 (34.9)	12.8 (51.9)	<0.001
		O/E ratio	9.6 (63.0)	8.4 (59.8)	18.4 (82.4)	<0.001
DM short term complication		Admission rate (%)	1.0 (8.9)	1.2 (9.7)	0.0 (0.0)	0.563
		O/E ratio	0.6 (5.2)	0.7 (5.7)	0.0 (0.0)	0.554
DM long term complication		Admission rate (%)	13.0 (45.7)	11.5 (41.8)	27.3 (71.4)	<0.001
		O/E ratio	9.0 (39.7)	8.0 (37.2)	17.9 (57.8)	<0.001
LOS for ACSCs admission		LOS (days)	10.8 (44.3)	10.4 (39.4)	11.2 (49.2)	0.118
		O/E ratio	11.1 (71.2)	8.6 (57.6)	28.1 (129.8)	<0.001

Abbreviations: ACSCs, ambulatory care sensitive conditions; CCI, Charlson comorbidity index; CHF, congestive heart failure; COPD, chronic obstructive pulmonary disease; DM, diabetes mellitus; LOS, length of stay; NHIS, National Health Insurance Service; OOP, out-of-pocket; O/E ratio, ratio of observed-to-expected ACSC admission rate or LOS for ACSCs admission; PCP, primary care provider; SD, standard deviation; SMDGs, simple or minor disease groups.

Note: p-values were calculated by χ^2^ test for categorical variables and *t* test for continuous variables.

* Expected ACSC admission rates and LOS for ACSCs admission were produced by the Poisson regression adjusting for age, sex, and CCI score.

### Primary care provider clusters: potential vs. specialized

Two-step cluster analysis results classified 14,710 (52.9%) private clinics into potential PCP cluster and 13,087 (47.1%) into specialized PCP cluster (silhouette coefficient = 0.3). Most of internal medicine, family medicine, pediatrics, otorhinolaryngology, and general practitioner, and around a half of thoracic and cardiovascular surgery and general surgery were classified as potential PCP cluster, and most of psychiatry, neurology, obstetrics and gynecology, orthopedics, neurosurgery, plastic surgery, ophthalmology, anesthesiology, rehabilitation medicine, dermatology, urology, and radiology were classified as specialized PCP cluster. Potential PCPs included mostly clinics with specialties of internal medicine (49.2%), family medicine (5.8%), pediatrics (12.6%), and general practitioner (11.1%), in total comprising 78.6%. Half of the other 21.4% specialties comprised otorhinolaryngology (11.3%) and a quarter comprised orthopedics (5.5%). Private clinics with surgical specialties were mostly categorized into specialized PCPs, followed by orthopedics (23.3%), dermatology (17.9%), ophthalmology (11.4%), obstetrics and gynecology (10.6%), urology (6.9%), and psychiatry (6.7%) clinics, together comprising 76.8%.

Private clinics belonging to potential PCP cluster, as compared to specialized PCP cluster, were located in a non-metropolitan area (29.2%) and tended to care for greater number of patients who were male (44.5%), aged ≤19 years (24.3%), and ≥65 years (15.2%), as well as those who had CCI score of ≥1 (15.4%), SMDGs (92.9%), and a high number of accompanied SMDGs (2.9); these private clinics also had a greater number of total visits and visits per patient, a higher proportion of visits to a specific clinic (36.3%) and a lower number of additional clinics visited (3.3) (Table 2).

Results of external validation of clusters showed that the potential PCP cluster had no difference in OOP cost compared to specialized cluster while its total claim income was 273,376 USD, which was 23,398 USD higher than the specialized cluster. In patients with at least one of 6 ACSCs, the proportion of patients admitted to a hospital because of ACSC was 13.5%, significantly lower than the admission rate of 44.5% in clinics in the specialized PCP cluster. Although there was no difference in admission rates for COPD, CHF and diabetes short-term complication, admission rates for asthma, diabetes without complication, and diabetes long-term complication were significantly low. The average LOS for ACSCs admission of visiting patients was 0.8 days shorter in potential PCP than in specialized PCP clusters, but this difference was not statistically significant. After adjusting for age, sex, and CCI score, O/E ratios of admission rates for all 6 ACSCs and asthma showed borderline significant differences among the PCP clusters; however, the difference in O/E ratios of admission rates for DM and DM long-term complication and LOS for ACSCs admission was statistically significant (Table 2).

Stability of PCP clusters was cross-validated using discriminant analysis. In a discrimination model, on entering all clustering variables as independent variables, significant differences were observed between PCP clusters (Wilks’ lambda = 0.196; p < 0.001). In all clustering variables, except percentage of Medical Aid patients, there was a difference between PCP clusters at a significance level of 1%. About 80.4% of variations between PCP clusters were explained by clustering variables. Standardized canonical discriminant function coefficient of the number of SMDGs per patient was -1.16, with influence of discriminant function as the biggest contribution. Coefficients of 20 percentages of patients with each SMC varied from 0.01 to 0.95, in which discriminant coefficient of patients with SMCs related to eye disease, skeletal disease, skin disease 1, female disease, and common disease in primary care was higher than 0.5. Percentage of patients with SMDGs was -0.66 and it was the 4^th^ most important discriminating factor out of the 33 clustering variables. Coefficients of specialty and location of private clinics, percentages of patients aged ≤19 years, patients aged ≥65 years, and Medical Aid patients, number of additional clinics visited, proportion of visits to specific clinic, number of patients with SMDGs, and number of visits per patient were all less than 0.2, having minimal contribution in explaining the differences between two groups. As a result of applying the discriminant function equation on the holdout samples, potential PCP group was predicted at 97.1% and specialized PCP group at 96.0%, showing an overall high predictive power of 96.6%.

**Fig 1:**
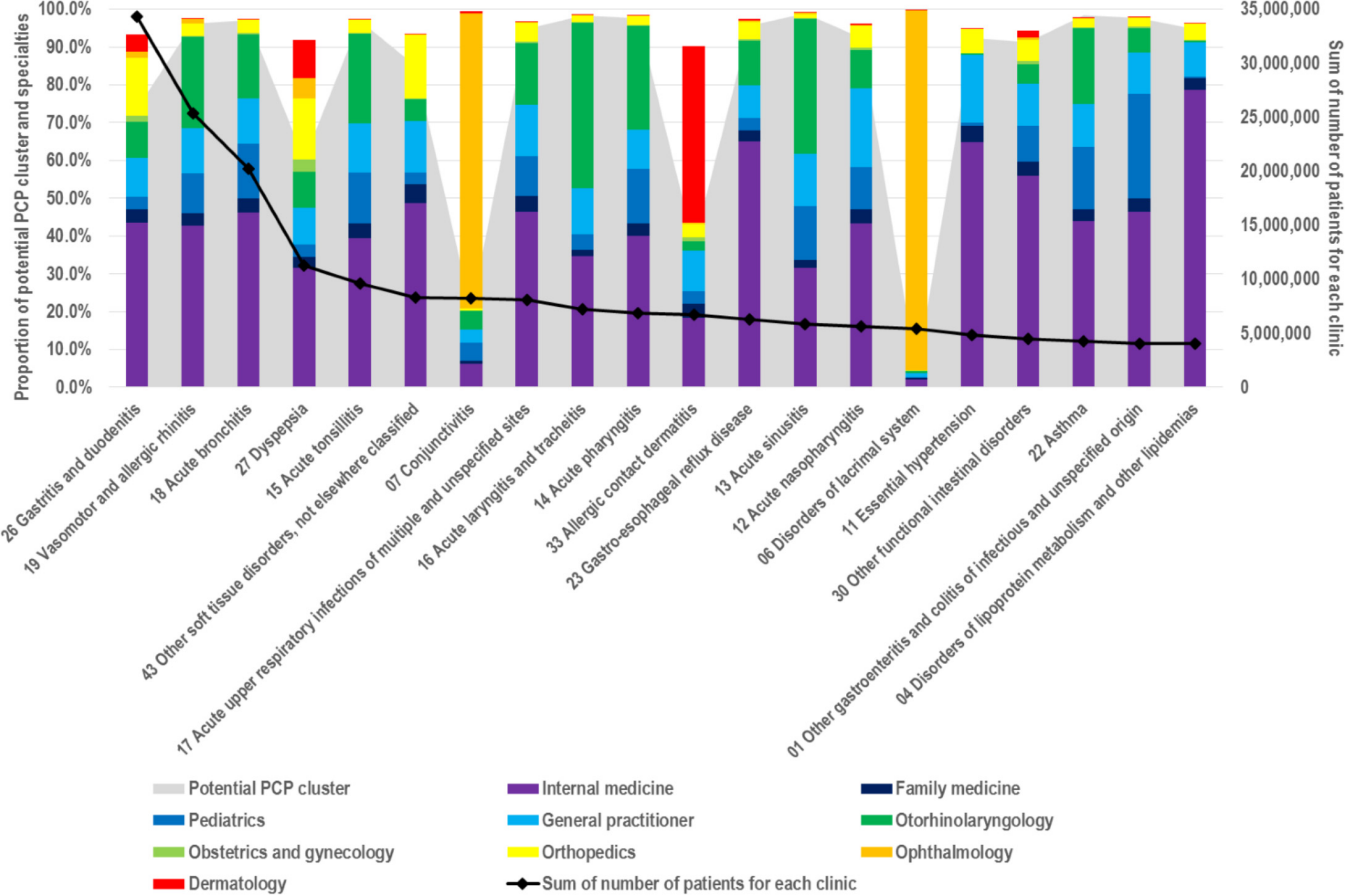
Twenty most common simple or minor disease groups and proportions of potential PCP clusters and specialties. Abbreviations: PCP, primary care provider.

PCP cluster variable for private clinics was merged into the data of 3,304,445 patients to evaluate the population size of patients treated by private clinics in the potential PCP cluster. By applying a sample weight of 33.3, the sum of the number of patients for each clinic was 110,147,042 and the sum of the number of patients with SMDGs for each clinic was 92,030,093, of which 56.8% and 63.6%, respectively, were treated by clinics in the PCP cluster. Based on 20 SMDGs with high number of patients, PCP clusters and distribution of 9 specialties serving many patients were calculated. The number of patients with gastritis and duodenitis was the highest at 34,325,982 (31.2%), followed by those with vasomotor and allergic rhinitis at 25,339,277 (23.0%), acute bronchitis at 20,267,994 (18.4%) and dyspepsia at 11,242,452 (10.2%). Thirteen of the most frequent SMDGs except conjunctivitis, allergic contact dermatitis, and disorders of lacrimal system were mostly treated by the potential PCP cluster. Excluding three disease groups treated more frequently by specialized PCP cluster and acute laryngitis, tracheitis, and acute sinusitis treated mostly by otorhinolaryngology departments, the specialty of treatment most commonly received by frequent SMDGs patients was internal medicine (31.6%–78.6%). Patients with vasomotor and allergic rhinitis, acute bronchitis, acute tonsillitis, acute upper respiratory infections of multiple and unspecified sites, and asthma were treated under otorhinolaryngology, gastritis and duodenitis, dyspepsia, and other soft tissue disorders, and patients who were not classified elsewhere were most frequently treated under orthopedics followed by internal medicine (Fig 1).

## Discussion

This study aimed to identify private clinics that have a potential to perform the role of PCP in a primary care setting in Korea where private specialists are dominant. To achieve this, a two-step cluster analysis using characteristics of private clinics was performed. The result classified more than a half of private clinics into a potential PCP cluster. In general, these were private clinics with specialties considered to be those of primary care physicians [27, 43, 44] and were more likely to be located in non-metropolitan areas than specialized PCPs were. Further, compared to specialized PCPs, they had a higher percentage of pediatric and geriatric patients, patients with greater disease severity, a higher percentage of patients with complex comorbidities or with SMDGs, a higher number of patients and visits, and the same or higher quality of primary care.

Private clinics of internal medicine, family medicine, pediatrics, and general practice, all considered primary care specialties, were mostly classified into the potential PCP cluster. However, 14.2% of family medicine specialists and 29.8% of general practitioners showed practice behavior similar to that of specialized PCPs. Further, 7.9% to 51.8% of thoracic and cardiovascular surgery, general surgery, neurology, orthopedics, anesthesiology, and rehabilitation medicine clinics could not focus on their specialties and rather followed practice behaviors of potential PCPs (Table 2). The latter case may point to a generalist role or “hidden system of primary care” of specialized PCPs [45]. However, it can also be viewed as a reflection of the efforts by some clinics with less market competition to secure patients and provide relatively more profitable services in an environment with high competition among the medical institutions [21, 22], which has been created in Korea due to low remuneration and a healthcare delivery system that has not yet been established [13]. Although clustering of private clinics was performed using various combinations of clustering variables and clustering methods, the quality of the PCP cluster remained around fair (silhouette coefficient = 0.3). This may be explained by convergence of practice behavior as a result of competition among primary care practices in securing patients and the subsequently blurred distinction in characteristics of patients treated by different specialties. Although there were a few cluster models with silhouette coefficient higher than 0.5, those models classified private clinics into heavily skewed groups (99% vs. 1%). They would not correspond to the objective of this study, which was to find potential PCPs in the primary care setting dominated by specialists with similar practice behavior.

However, private clinics seemed to be appropriately classified into PCP clusters. Compared to specialized PCPs, potential PCPs treated a higher number of vulnerable patients with multiple healthcare needs more frequently and were more likely to be located in a non-metropolitan area. Because of low birth rates and rapid aging of the population in Korea, there is an urgent need for reduction in socio-economical burden due to non-communicable diseases in elderly people and management of health in children for conservation of reproductive populations [8, 17]. Compared to metropolitan areas, the proportion of people aged ≥ 65 years was 1.5 times higher (15.7% vs. 10.7%) and the proportion of Medical Aid beneficiaries was 2.3 times higher (4.7% vs. 2.1%) in non-metropolitan areas [46]. These aged and deprived populations were at higher risk of having multi-morbidities in Korea [47]. Potential PCPs are more likely to satisfy such social needs compared to specialized PCPs. Analyses of differences in ACSC admission rates and LOS for ACSC admission, which were not clustering variables, revealed that outcomes of care were the same or more favorable in potential PCPs than in specialized PCPs. This was similar to results from previous studies that showed a higher quality of primary care provided by typical primary care physicians such as specialists in internal medicine, family medicine, and pediatrics that comprise potential PCPs [4–6, 19, 26, 45, 48, 49].

The proportion of visits to a specific clinic and number of additional clinics visited were also more favorable among potential PCPs than among specialized PCPs. Among the annual total visits to private clinics, in average, 36.3% were visits to a private clinic associated with potential PCPs; this percentage was 7.7% point higher than visits to specialized PCPs. The number of additional clinics visited was smaller among the potential PCPs than among the specialized PCPs; however, the difference was 0.5. Although these differences were statistically significant, even patients who utilized the potential PCPs saw other non-usual providers, accounting for as much as 63.7% of their annual total visits, and may have visited more than three additional clinics for their various conditions. This may imply that even if potential PCPs can act as usual providers better than specialized PCPs, coordination and integration among potential and specialized PCPs are needed to provide improved comprehensive and longitudinal care.

Regarding average annual total claim cost of clinics, potential PCPs had higher costs than specialized PCPs did, which can be attributed to the high number of patients and visits at potential PCP clinics. Average total claim cost per visit and average OOP cost per visit were lower for potential PCPs (15.8 USD, 3.7 USD) than for specialized PCPs (21.5 USD, 5.5 USD). This may be attributed to differences in profitability because of patient characteristics in different PCP clusters, and not because of efficient service provision of potential PCPs. It is well known that primary care reduces overall healthcare costs by decreasing preventable or unnecessary healthcare utilization [4–6]. However, under the fee-for-service payment system with low remuneration in Korea [50], healthcare providers are incentivized to increase the frequency of patient visits. In fact, the number of doctor consultations per person in Korea is 2.2 times that of the OECD average, and the highest in all OECD countries [8]. Therefore, as a potential PCP has less income per patient visit than a specialized PCP [21], there is a stronger incentive to increase the number of visits, which may have contributed to these results. This suggests that primary care in Korea is not successful in performing its role of rationally distributing healthcare resources appropriately for patients [7, 17]. Imbalance in geographic distribution of healthcare providers is due to several complex factors, such as the relative unattractiveness of the rural areas, insufficient compensation for physicians, and lack of professional prestige [51]. Along with these factors, differences in profitability between metropolitan and non-metropolitan areas may partly have influenced the geographic distribution of potential PCPs in private sector dominant and competitive primary care settings in Korea. Total claim cost per patient of the specialized PCPs was higher in metropolitan area (69.2 USD) than in non-metropolitan area (67.7 USD), and that of the potential PCPs was higher in non-metropolitan area (67.1 USD) than in metropolitan area (63.1 USD).

The most important factor in explaining variations between PCP clusters was the number of SMDGs per patient. The proportion of patients with SMDGs commonly treated in private clinics and percentage of patients with SMDGs were also important discriminant factors. In addition, other institutional, patient, or utilization characteristics of private clinics had minimal effects on classification of PCP clusters. These results suggest that the number of patients with common and complex health needs in the Korean population managed by private clinics can be used as a criterion in identifying potential PCPs in the primary care setting of Korea in which private clinics have little to offer in distinguish themselves from other private clinics. Many governments of industrialized countries have implemented reforms based on the Chronic Care Model for strengthening primary care to cope with aging populations, focusing on integrated and patient-centered care for patients with complex health needs [52]. Although Korea is experiencing more severe demographic changes than other industrialized countries [17], its primary care system is still underdeveloped [7]. The potential PCP-identifying criteria reported in this study could help define PCPs that can help the Korean healthcare system respond to this challenge.

This study has several limitations. First, as the specialty of private clinics is self-assigned based on claim data, it may not correspond with the true specialty of the clinics. However, in approximately one third of the private clinics, the physicians run clinics or practice medicine in areas different from their actual specialty area [21]. Self-assigned specialty is a variable that reflects actual practice behavior as it is based on the information provided for cost claims, in which private clinics themselves fill out and submit information on which specialty care was provided. Therefore, it was a variable that allowed for classification of PCP clusters reflecting the actual practice behavior of private clinics. Second, PCP clusters were classified based on the level of primary care practice, not on the physicians practicing primary care. Clustering of private clinics was performed because if a preferred physician system that cares for registered patients and plays a role in gatekeeping is to be implemented in Korea, it will be run at the level of private clinics, and not by individual physicians. Third, SMDGs were proxy indicators for comprehensiveness attributes of primary care. The concept of comprehensiveness includes not only treating various common problems associated with diseases, but also disease prevention, early detection, and health promotion [11, 53]. SMDGs consist of 52 disease groups commonly seen in primary care in Korea, where the government recommends treatment in private clinics over that in hospitals [32]. It is insufficient to embrace the entire concept of comprehensiveness. However, since SMDGs are meaningful as a policy tool of the government to strengthen primary care, they were utilized as variables to identify potential PCPs. Fourth, we were not able to include other attributes of primary care such as coordination of care, personalized care, or context of family and community in our analysis. Although the proportion of visits to a specific clinic and number of additional clinics visited were included in the analysis, they were proxies to assess continuity of care at the provider level, which were revised measures of continuity of care at the individual patient level. In Korea, except for programs that provide financial incentives for providing a regular source of care for patients with chronic diseases or some Medical Aid patients [13, 26], there is no official gatekeeping system. Hence, this study focused on comprehensiveness rather than continuity of care. However, there is a need to identify potential PCPs by including indicators related to other attributes of primary care as clustering variables in future studies.

Korea is currently experiencing rapid and concerning demographic changes. The speed of aging of the population is the highest and birth rate is the lowest among all OECD countries [7]. To improve the responsiveness and sustainability of the healthcare system in response to growing health care needs, there is a pressing need for strengthening the primary care system [54]. However, over the last two decades, the government of Korea has not taken specific steps to strengthen the primary care system. One of the reasons was that it was difficult to define policy targets without first ascertaining the definition and criteria of PCP and the process of assigning the role of PCP. For strengthening of primary care, procuring a sufficient number of PCPs who will deliver adequate primary care will be important in addition to implementation of various other policies [55]. The number of practicing physicians in Korea is two thirds of the average value for OECD [8]. Fortunately, the rate of increase in number of physicians is high [44], but they lack systematic training to carry out PCP responsibilities (under-qualification) [18] and provide primary care outside their specialty (mis- or over-qualification). Without generating an appropriate system of specialist-centered primary care professionals, it will be difficult to solve the problems of PCP shortage. Further, training of new PCPs excluding the existing practicing physicians is not a feasible alternative. Therefore, this study identified potential PCPs while accounting for the circumstances of primary care in Korea and suggested the identifying criteria for PCPs. Although this study is only the first step in strengthening primary care in Korea, it will provide useful information for formulation of a primary care strengthening policy to policy makers in Korea as well as other countries with similar specialist-dominant primary care settings.
